# Geometry influences inflammatory host cell response and remodeling in tissue-engineered heart valves in-vivo

**DOI:** 10.1038/s41598-020-76322-9

**Published:** 2020-11-16

**Authors:** Sarah E. Motta, Emanuela S. Fioretta, Valentina Lintas, Petra E. Dijkman, Monika Hilbe, Laura Frese, Nikola Cesarovic, Sandra Loerakker, Frank P. T. Baaijens, Volkmar Falk, Simon P. Hoerstrup, Maximilian Y. Emmert

**Affiliations:** 1 Institute for Regenerative Medicine (IREM), University of Zurich, Wagistrasse 12, 8952 Schlieren, Switzerland; 2 Wyss Translational Center Zurich, University and ETH Zurich, Zurich, Switzerland; 3 Institute of Veterinary Pathology, University of Zurich, Zurich, Switzerland; 4 Department of Cardiothoracic and Vascular Surgery, German Heart Center Berlin, Berlin, Germany; 5 Department of Health Sciences and Technology, Swiss Federal Institute of Technology, Zurich, Switzerland; 6 Department of Biomedical Engineering, Eindhoven University of Technology, Eindhoven, The Netherlands; 7 Institute for Complex Molecular Systems, Eindhoven University of Technology, Eindhoven, The Netherlands; 8 Department of Cardiovascular Surgery, Charité Universitätsmedizin Berlin, Berlin, Germany

**Keywords:** Biotechnology, Tissue engineering, Cardiovascular diseases, Valvular disease

## Abstract

Regenerative tissue-engineered matrix-based heart valves (TEM-based TEHVs) may become an alternative to currently-used bioprostheses for transcatheter valve replacement. We recently identified TEM-based TEHVs-geometry as one key-factor guiding their remodeling towards successful long-term performance or failure. While our *first-generation* TEHVs, with a simple, non-physiological valve-geometry, failed over time due to leaflet-wall fusion phenomena, our *second-generation* TEHVs, with a computational modeling-inspired design, showed native-like remodeling resulting in long-term performance. However, a thorough understanding on how TEHV-geometry impacts the underlying host cell response, which in return determines tissue remodeling, is not yet fully understood. To assess that, we here present a comparative samples evaluation derived from our *first-* and *second-generation* TEHVs. We performed an in-depth qualitative and quantitative (immuno-)histological analysis focusing on key-players of the inflammatory and remodeling cascades (M1/M2 macrophages, α-SMA^+^- and endothelial cells). *First-generation* TEHVs were prone to chronic inflammation, showing a high presence of macrophages and α-SMA^+^-cells, hinge-area thickening, and delayed endothelialization. *Second-generation* TEHVs presented with negligible amounts of macrophages and α-SMA^+^-cells, absence of hinge-area thickening, and early endothelialization. Our results suggest that TEHV-geometry can significantly influence the host cell response by determining the infiltration and presence of macrophages and α-SMA^+^-cells, which play a crucial role in orchestrating TEHV remodeling.

## Introduction

Current transcatheter bioprosthetic heart valve replacements are based on glutaraldehyde-fixed animal-derived materials and are therefore associated with long-term structural degradation and limited durability^1^. Additionally, such bioprostheses are associated with major limitations (i.e. risk for infection and thrombosis) as they lack the fundamental properties of native tissues such a native-like remodeling potential, and importantly also the capacity for growth^1^.

Heart valve tissue engineering approaches aim at the creation of biological substitutes capable of integration, native-like remodeling, and growth^2,3^. In this context, acellular tissue-engineered heart valves (TEHVs) for in-situ regeneration are particularly interesting due to their off-the-shelf availability and scalability compared to classical autologous tissue-engineered substitutes^2–5^. Notably, acellular TEHVs based on decellularized in-vitro-grown tissue-engineered matrices (TEM) showed promising results as pulmonary valve replacements in a translational animal model using transcatheter implantation techniques^6,7^.

**Figure 1:**
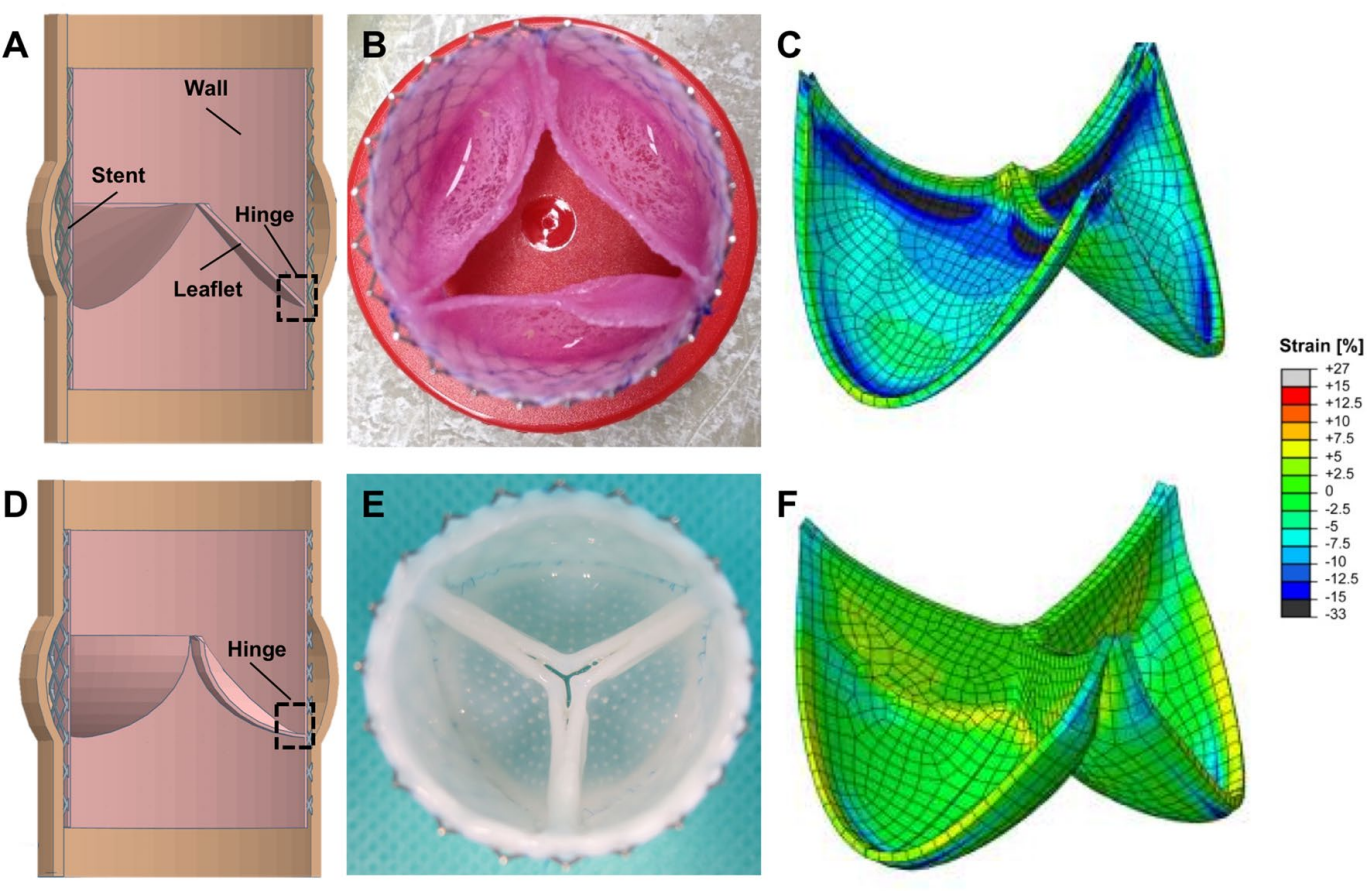
First- and second-generation TEM-based TEHVs. (**A**) Schematic of the cross-section of a first-generation TEHV comprising the nitinol stent, the valve wall, leaflet, and the characteristic acute angle of the hinge region (dotted square)^6^. Image was created using the software Tinkercad under *Creative Commons license Non-Commercial 3.0*. (**B**) Macroscopic appearance of a first-generation TEHV after in-vitro culture. (**C**) In the first-generation TEHV design the leaflets are exposed to compressive strain (dark blue areas in the coaptation and hinge regions) in radial direction. Image was re-adapted from Sanders et al.^8^ under the *Creative Commons license 4.0*. (**D**) Schematic of the cross-section of a second-generation TEHV^7^, characterized by a more profound hinge region (dotted square). Image was created using the software Tinkercad under *Creative Commons license Non-Commercial 3.0*. (**E**) Macroscopic appearance of a second-generation TEHV after in-vitro culture performed with an insert to impose valve geometry^8^. (**F**) In second-generation TEHV design radial tissue compression was limited to the hinge region, thereby not affecting the leaflets coaptation area. Image was re-adapted from Sanders et al.^8^ under the *Creative Commons license 4.0*.

Initially we manufactured our TEM-based TEHVs using a simple, non-physiological valve geometry (to which we refer to as *first-generation* TEHVs in this manuscript; Fig. 1A–C) for transcatheter pulmonary valve replacement (TPVR) in a preclinical sheep model^6^. Despite the promising valve performance in the first week after implantation, such *first-generation* TEHVs showed progressive valvular insufficiency caused by leaflet-wall fusion phenomena and leaflet shortening over time^6^. Therefore, we recently used a computational modeling (in-silico) approach to achieve an analytical physiological-like design, to which we refer to as *second-generation* TEM-based TEHV (Fig. 1D–F)^7^. Such TEHVs comprise an optimized strain distribution profile within the leaflets, in order to prevent tissue compaction and endure functional remodeling, thereby enabling long-term performance^7^. *Second-generation* TEHVs were manufactured using the same tissue culture processes as for our *first-generation* TEHVs, including the same cell source (ovine myofibroblasts), scaffold composite (polyglycolic acid coated with poly-4-hydroxy butyrate), and bioreactor technology^6,7^.

Remarkably, after TPVR in sheep, *second-generation* TEHVs demonstrated clinical-grade and preserved long-term performance with the absence of relevant regurgitation and only minimal leaflet shortening, as predicted by the computational models^7^. While both generations of TEM-based valves showed profound and continuous remodeling potential over time, comprising host cellular infiltration, neo-matrix deposition, and endothelialization, *first-generation* TEHVs presented with maladaptive remodeling phenomena (i.e. leaflet shortening and thickening) starting several weeks post-implantation. To the contrary, *second-generation*, computational modeling-inspired TEHVs, exhibited tissue remodeling towards native-like configurations.

So far, previous studies have identified two important parameters that are relevant in the remodeling process of TEHVs. First, the geometry^7,8^, which influences the stress and strain distribution in the leaflets thereby determining long-term valve functionality. Second, the presence and distribution of α-smooth muscle actin positive (α-SMA^+^) cells within the valves^9^, which if over-expressed in the leaflets, induce leaflet retraction, leading to dysfunction and ultimate valve failure^9^. However, despite the identification of these predictors, the impact and role of inflammatory cells in the remodeling processes, that determines failure^6^ or success^7^ of the implanted TEM-based TEHVs is not yet fully understood. Hence, the in-depth understanding of the underlying inflammatory processes, especially during the early stages of the remodeling process, are mandatory to determine and guide the outcome thereby enabling safe clinical translation of TEM-based TEHVs.

Inflammatory host cells such as monocyte-derived naïve macrophages can polarize in response to the local mechanical and biochemical environmental stimuli and/or implanted biomaterial, and can differentiate into a variety of cells that have specific biological functions^10^. The extreme ends of this polarization range are referred to as the pro-inflammatory macrophage 1 (M1) and the anti-inflammatory macrophage 2 (M2) type^11^. While M1 macrophages are typically present during an inflammatory phase, M2 cells are primarily active during reparative and constructive remodeling^11^.

Macrophages play a pivotal role in the initiation and perpetuation of inflammation and remodeling processes, thereby determining either the chronicity or the resolution of the inflammatory process—and this has been demonstrated also for tissue-engineered implants such as vascular grafts^12–18^. In these studies, M2 macrophages were shown to help the recruitment of tissue remodeling cells (e.g.: smooth muscle cells, endothelial cells (ECs), and other immune cells) by expressing anti-inflammatory cytokines, finally leading to adaptive and functional remodeling^13,19–23^. To the contrary, the constant presence of the M1 phenotype, which gives rise to a chronic inflammation and scar tissue formation, will finally result into a negative or maladaptive remodeling^11^.

In this study, we assessed the role and impact of inflammatory host cells on the (mal)-adaptive remodeling processes observed in the hinge area of TEM-based TEHVs. To achieve this, we performed an independent, in-depth qualitative and quantitative (immuno-)histological analysis in tissue samples derived from the hinge area of our previous *first-*^6^ and *second-generation*^7^ TEHV preclinical studies (Fig. 1). We analyzed key cellular players of the inflammatory and remodeling cascades such as M1 and M2 macrophages, α-SMA^+^ cells, and ECs. We focused our analysis on the hinge region of the TEHVs as this area is exposed to a maximum degree of mechanical strain^24^, which is known to be a strong regulator of valvular cell behavior and differentiation^25^,that in turn strongly influences the (mal)adaptive remodeling phenomena in TEHVs.

## Results

### *First-generation* TEHV analyses

#### Qualitative evaluation of inflammatory host cell infiltration

**Figure 2:**
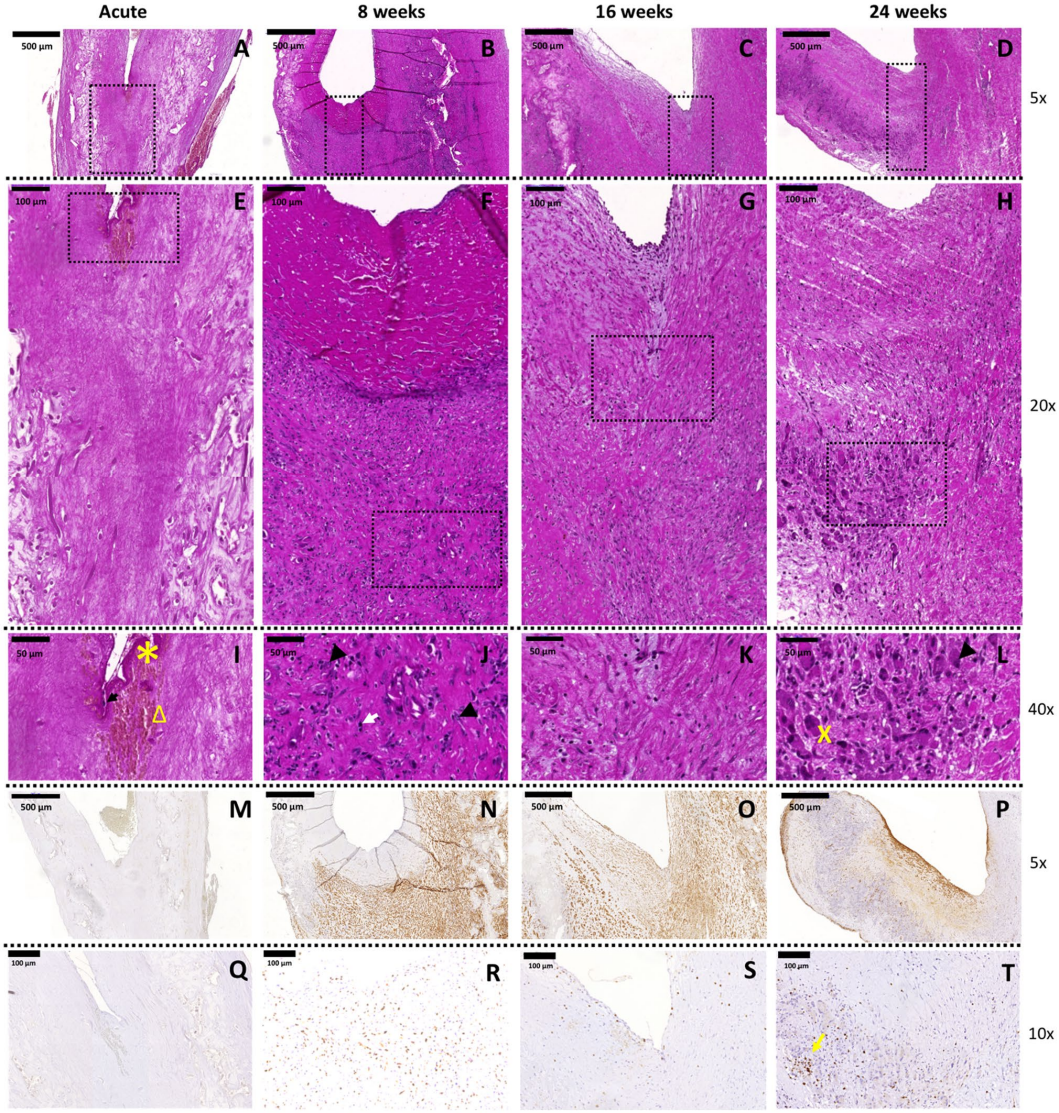
Hinge region of *first-generation* TEHVs at different time-points. Representative images of the H&E staining performed on the cross section of the valve. (**A**–**D)** 5 × magnification pictures of the hinge region of the valve (scale bars 500 μm). (**E**–**H**) 20 × magnification pictures of the hinge region marked by the dotted rectangles in panels (**A**–**D**) (scale bars 100 μm). (**I**–**L**) 40 × magnification images of the hinge region marked by dotted rectangles in panels (**E**–**H**) (scale bars 50 μm). Presence of contractile cells is detected with immunohistochemical images at 8 (**N**), 16 (**O**), and 24 (**P**) weeks. α-SMA^+^cells are already detected in the hinge area at the earliest time point considered and, over time, these cells are also present in the leaflet. Acute control valve (**M**) does not present α-SMA^+^cells. Scale bars: 500 μm, zoom: 5 × . Presence of T-lymphocytes is detected with immunohistochemical images at 8 (**R**), 16 (**S**), and 24 (**T**) weeks. Acute control valve (**Q**) does not present T-lymphocytes. T-lymphocytes cluster around polymer remnants or in follicles (yellow arrow). Scale bars: 100 μm, zoom: 10 × . In panel I arrow points at leukocytes, star symbol indicates fibrin, and triangle marks erythrocytes. In panel (**J**) arrow heads point at macrophages and the white arrow indicates eosinophils. In panel (**L**), the cross symbol shows multinucleated giant cells and the arrowhead macrophages.

H&E and MG staining showed abundant cell infiltration over time throughout the valve (Fig. 2 and Supplementary Fig. 1). In the explanted TEHVs from the acute animals, no cellular infiltration within the valve tissue was visible (Fig. 2 A,E,I); the only cells detected were those trapped in the fibrin thrombus (Fig. 2I, star symbol), i.e. erythrocytes (Fig. 2I, triangle) and leukocytes (Fig. 2I, arrow). At 8 weeks, cells were predominantly present in the wall and hinge regions of the valve (Fig. 2B,F). In addition, our analysis revealed a progressive colonization of cells in the hinge region (at 16 and 24 weeks), which further develop towards the leaflets (Fig. 2C,G,D,H). The analysis of the area surrounding the scaffold remnants in the hinge region showed the presence of fibrous tissue of heterogeneous thickness, with eosinophilic cells containing a multilobed nucleus (Fig. 2J, white arrow), multifocal ordered macrophages containing blue granules in the cytoplasm (Fig. 2J,L, arrow head), multinucleated giant cells (Fig. 2L, cross symbol), and isolated lymphocytes (small spherical nucleus with abundant dark staining) forming lymph follicles (Fig. 2J–L), all indicators of an ongoing chronic inflammatory response of the host towards the implanted TEHV and signs of incomplete polymer degradation at 24 weeks. Over time, α-SMA expression decreased in the wall region and gradually increased towards the hinge region and the leaflet (Fig. 2M–P). Abundant infiltration of CD3^+^ T lymphocytes was observed at the 8 weeks follow-up (Fig. 2R). After 16 (Fig. 2S) and 24 weeks (Fig. 2T), lymphocytes distribution changed from random distribution to multifocal, with clusters detected around polymer leftovers or in follicles. Presence of endogenous ECs was demonstrated via SEM and immunohistochemistry for CD31 and VWF (Supplementary Fig. 2) and was in line with the previously reported results for our *first-generation* TEHVs^6^. Interestingly, at 8 weeks inhomogeneous EC coverage was observed via SEM, CD31 and VWF staining (Supplementary Fig. 2A–E). In addition, we observed a large number of platelets (VWF^+^) entrapped in the pannus (Supplementary Fig. 2B). At later time-points (16 and 24 weeks), the valves demonstrated a confluent, homogenous and persistent spindle-shaped EC layer covering the entire surface (Supplementary Fig. 2F–O) as also confirmed by the immunohistochemistry for CD31 and VWF (Supplementary Fig. 2F–G and K–L). Importantly, the CD31^+^ ECs in the 16 weeks explants expressed the pro-thrombotic factor vWF (Supplementary Fig. 2G). Cutting artefacts may have prevented the complete depiction of ECs and platelets presence at 24 weeks for the CD31 and vWF stainings (Supplementary Fig. 2K–L), while ECs confluency could be confirmed by SEM imaging (Supplementary Fig. 2M–O).

#### Quantitative evaluation of macrophages in TEHV hinge area

**Figure 3:**
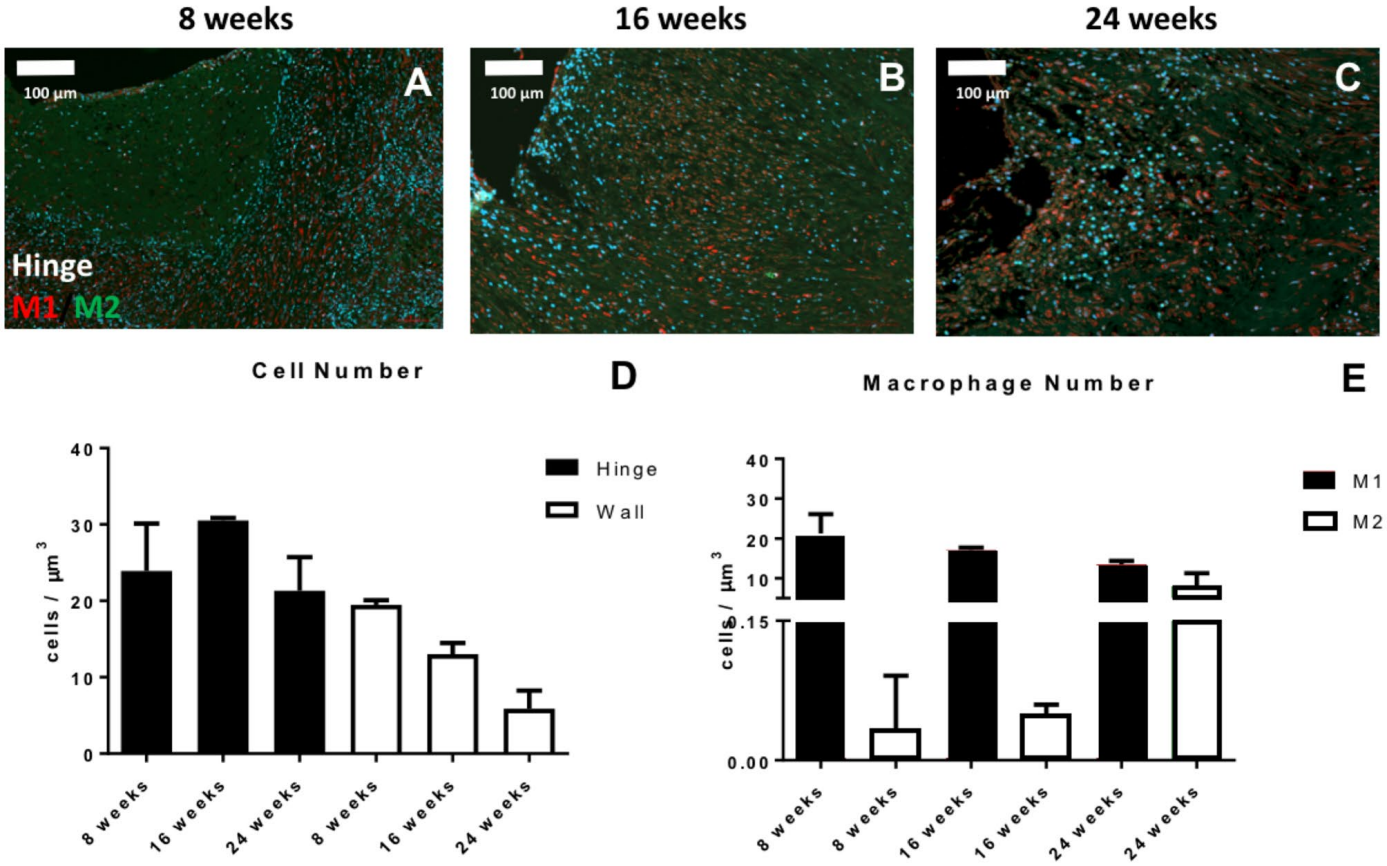
Analysis of cell phenotype for the hinge area of *first-generation* TEHVs. Immunofluorescence images at the hinge area stained for M1 (red) and M2 (green) macrophages at 8 (**A**), 16 (**B**), and 24 (**C**) weeks follow-up. (**D**) Total cell number quantification in the wall and hinge region of *first-generation* TEHVs is showing an abundant cell presence indicating a high ongoing inflammatory response. (**E**) Quantification of M1 and M2 macrophages in the hinge region at the different time-points considered shows a prevalence of M1 over M2 subpopulation, with the number of M1 macrophages gradually decreasing over time while M2 macrophages is increasing. For immunofluorescence pictures: scale bars: 100 μm, zoom 1 × . For every measurement, cell number is normalized per μm^3^.

Contrarily to what is observed for the control wall of TEHVs, the amount of cell infiltration in the hinge region, measured by cell nuclei quantification with DAPI-staining, did not gradually decreased over time (Fig. 3A–D). The number of stained M1 slightly decreased over time, whereas M2 macrophages dramatically increased beyond 8 weeks approaching the total number of M1 at 24 weeks. However, an overall prevalence of the M1 over the M2 phenotype is observed (Fig. 3E), suggesting an ongoing chronic inflammatory response in the hinge area of the TEHVs.

#### Morphological evaluation

**Figure 4:**
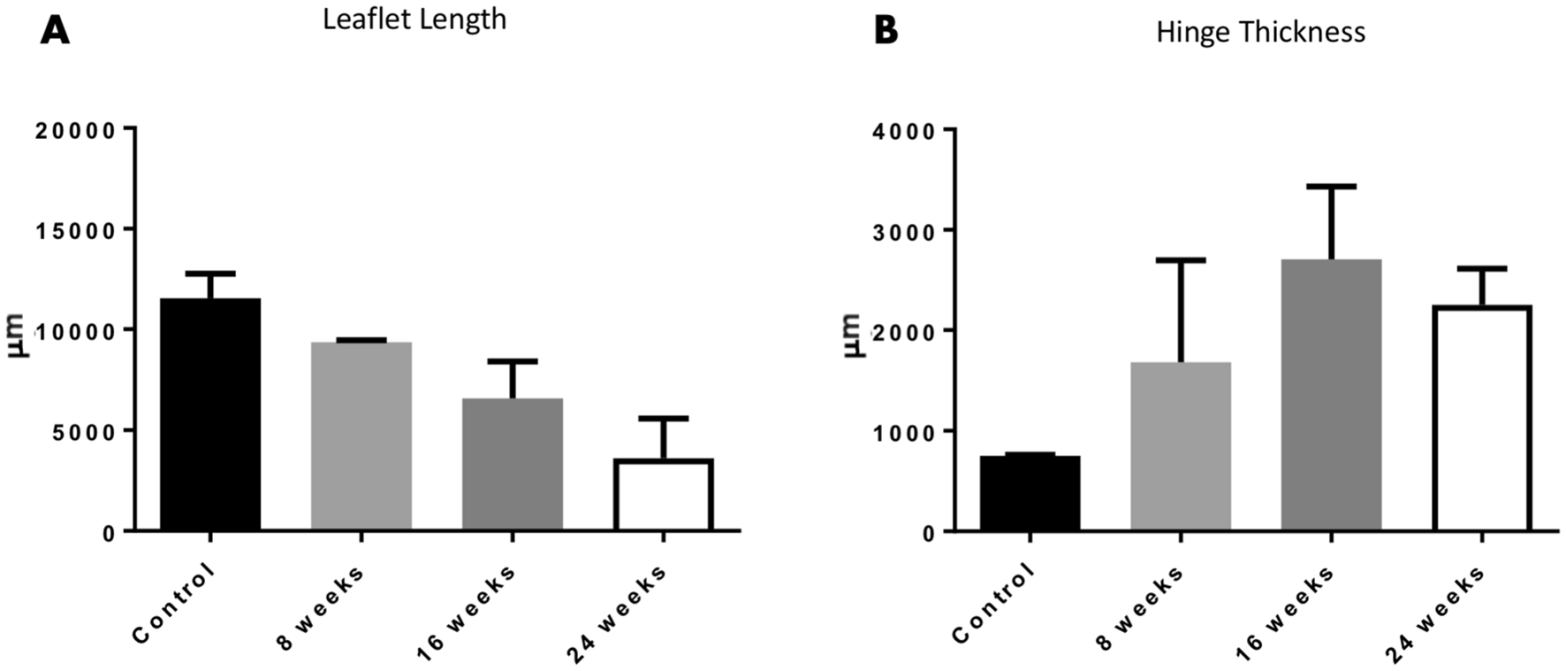
Tissue geometry of *first-generation* TEHVs. (**A**) Quantification of leaflet lengths from histological sections of *first-generation* TEHVs shows gradual leaflet shortening over time. (**B**) Quantification of hinge thickness from histological sections of *first-generation* TEHVs demonstrates increased values over time. Acute control TEHVs represent the initial leaflet length and hinge thickness of acutely-implanted valves. Measurements are expressed as average value ± standard deviation.

Morphological changes of the TEHV in terms of leaflet length and hinge thickness were evaluated for all available time points (Table 1). Compared to the leaflet length in the acute animals (controls) (Fig. 4A, black column), leaflets showed an initial shortening of 19% at the 8-weeks time point (Fig. 4A, grey bar) which then further progressed over time to a total shortening of approximately 70% at 24-weeks time point (Fig. 4A, white bar), which led to TEHVs functional failure. The hinge thickness increased over time with a total thickening of 66.5% at 24 weeks in-vivo when compared to the hinge thickness in the acute animals (controls) (Fig. 4B).

**Table 1:** Overview of the available samples for first- and *second-generation* TEHVs. (*) Unpublished data derived from an independent pilot study, in which TEHVs were cultured using the same culture conditions.

Geometry	Follow-up					
	Acute (= control)	8 weeks	16 weeks	24 weeks	52 weeks (= 1 year)	Total
First-generation	n = 2	n = 2	n = 4	n = 4	n.a	12
Second-generation	n = 2*	n = 3*	n.a	n.a	n = 3	8

### *Second-generation* TEHV analysis

#### Qualitative evaluation of inflammatory host cell infiltration

**Figure 5:**
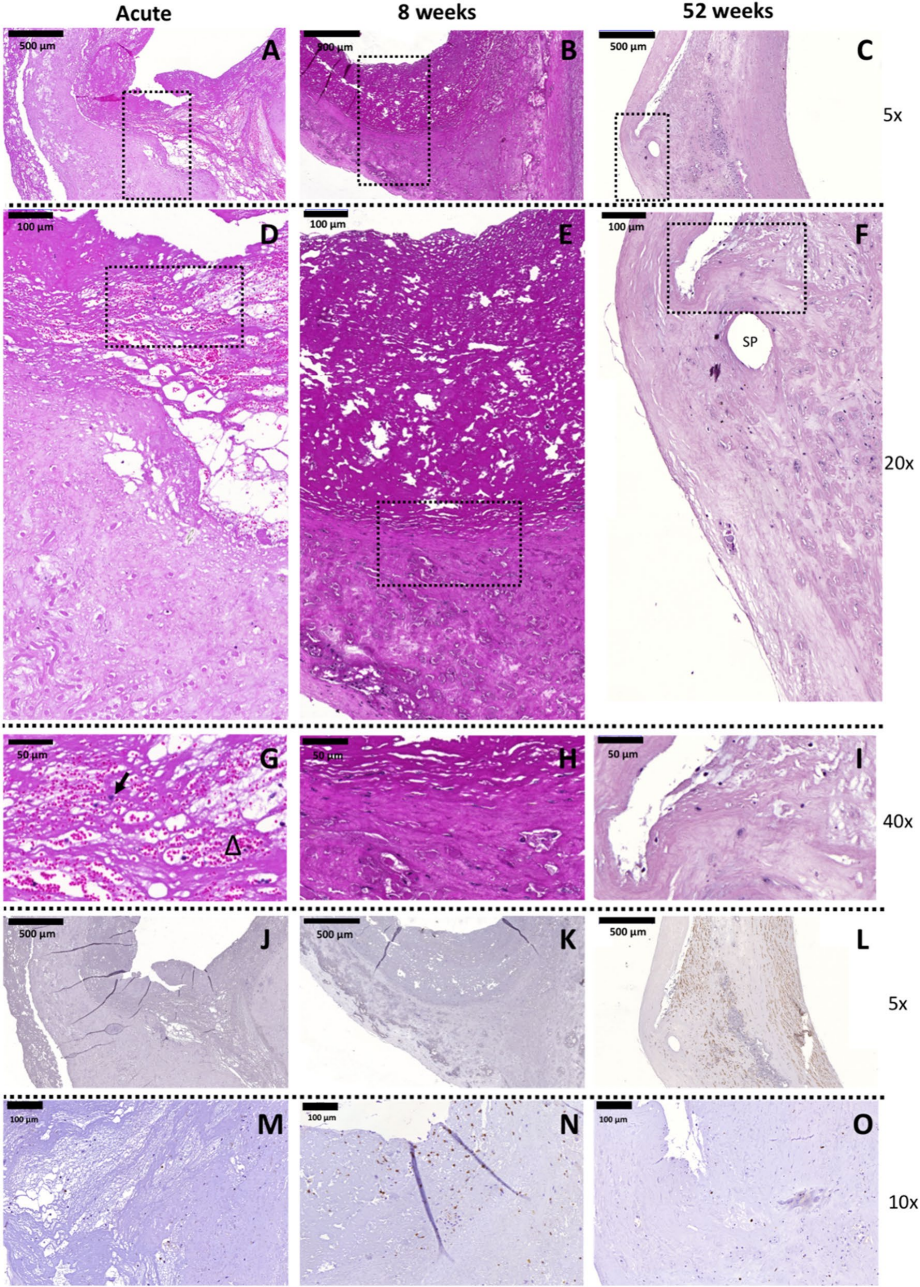
Hinge region of *second-generation* TEHVs at different time-points. Representative images of the H&E staining performed on the cross section of the valve. (**A**–**C)** 5 × magnification pictures of the hinge region of the valve (scale bars 500 μm). (**D**–**F**) 20 × magnification pictures of the hinge region marked by the dotted rectangles in panels (**A**–**C**) (scale bars 100 μm). (**G**–**I**) 40 × magnification of the hinge region marked by dotted rectangles in panels (**D**–**F**) (scale bars 50 μm). In panel (**F**), *SP* points at a hole left by a suture point. In panel (**G**), arrow points at leukocytes and triangle indicates erythrocytes. Immunohistochemical images of the hinge area of *second-generation* TEHVs stained for α-SMA at 8 weeks (**K**), and 52 weeks (**L**) follow-up. For this novel valve geometry, few to none α-SMA^+^ cells are detected in the hinge area or in the leaflet at the time-point considered. Acute control valve (**G**) does not present α-SMA^+^ cells. Scale bars: 500 μm, zoom: 5 × . Presence of T-lymphocytes is detected with immunohistochemical images at 8 (**N**) and 52 (**O**) weeks. Scale bars: 100 μm, zoom: 10 × .

H&E and MG staining showed few and sparse infiltrated cells in the hinge region at any time point considered (Fig. 5 and Supplementary Fig. 3). In the acute explant, few cells were detected in the fibrin clot (Fig. 5A,D,G): erythrocytes (Fig. 5G, triangle) and leukocytes (Fig. 5G, arrow). After 8 weeks in-vivo, cells were predominantly present in the wall regions of the valve, close to the native tissues (Fig. 5B,E). At 52 weeks follow-up, instead, cells were homogenously distributed in the different valve regions, with some cellular aggregates in the proximity of the few polymeric scaffold remnants (Fig. 5C,F). Cells expressing α-SMA were only detected in the wall area of the valve, suggesting the presence of a functional vascular wall. Independently of the time point considered, few to none α-SMA^+^ cells were detected in the hinge and leaflet region (Fig. 5J–L). In *second-generation* TEHVs, CD3^+^ T lymphocytes were overall less abundant compared to *first-generation* valves (Fig. 5M–O). Most of the CD3^+^ T lymphocytes were detected 8 weeks after implantation, accumulated in the pannus at the hinge area (Fig. 5N). After 52 weeks, only sporadic CD3^+^ cells were detected (Fig. 5O). Finally and in line with our previously reported results^7^, *second-generation* TEHVs were covered by a confluent EC layer already after 8 weeks, as shown by SEM imaging and immunohistochemistry (Supplementary Fig. 2). Most importantly, the confluent endothelium was retained up to 52 weeks.

#### Quantitative evaluation of macrophages in TEHV hinge area

**Figure 6:**
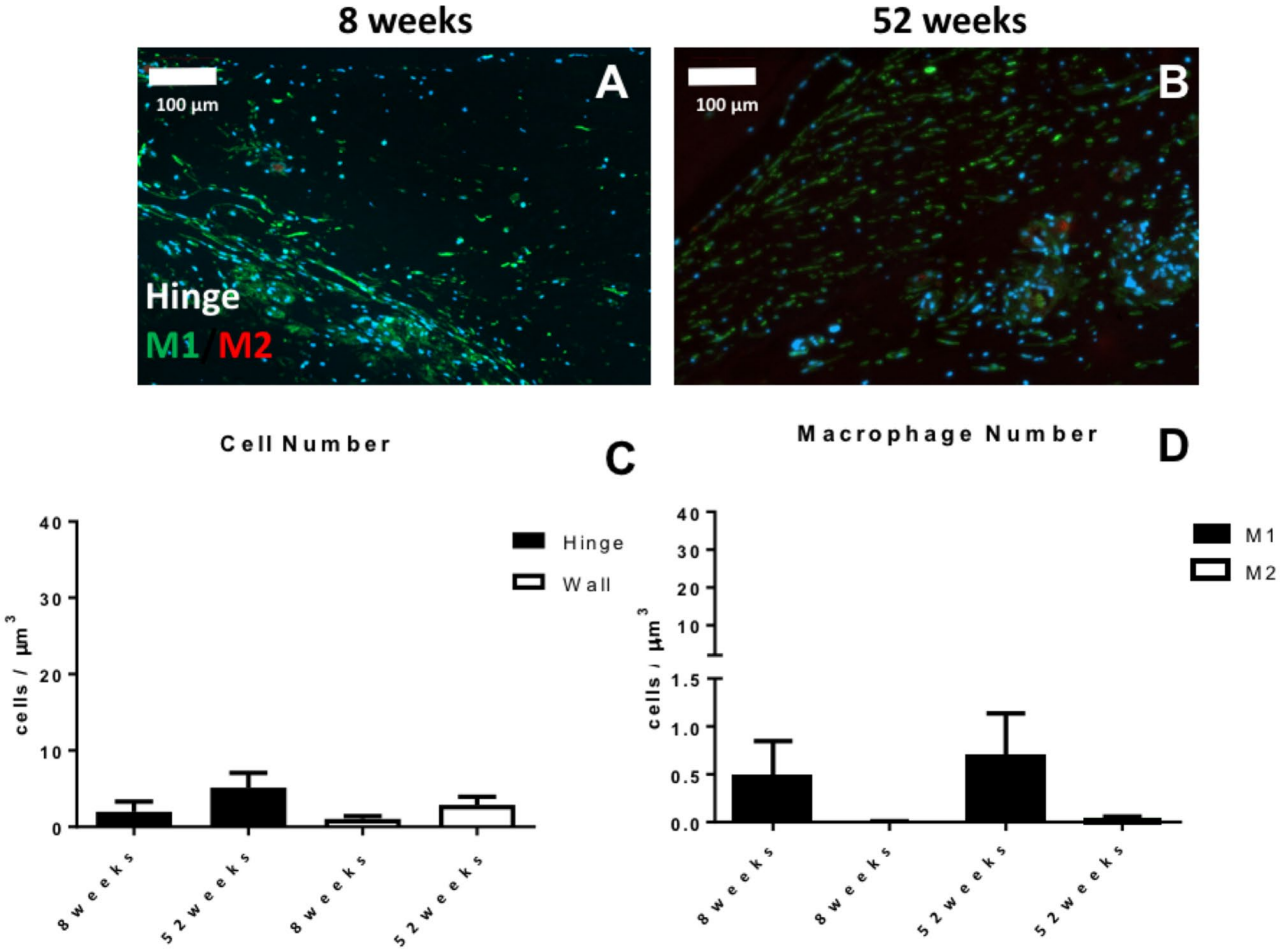
Analysis of cell phenotype for the hinge area of *second-generation* TEHVs. Immunofluorescence images at the hinge area stained for M1 (green) and M2 (red) macrophages at 8 weeks (**A**) and 52 weeks (**B**) follow-up. (**C**) Total cell number quantification in the wall and hinge region of *second-generation* TEHVs. (**D**) Quantification of M1 and M2 macrophages in the hinge region at the different time-points considered shows a mild prevalence of M1 over M2 positive cells. For immunofluorescence pictures: scale bars: 100 μm, zoom 1 × . For every measurement, cell number is normalized per μm^3^.

Quantification of the cell nuclei stained with DAPI (Fig. 6A–B) revealed a low number of cells in the hinge region at both 8 weeks and 52 weeks, values comparable to those of the wall region of the valve (Fig. 6C). Overall, a minor presence of macrophages, with a prevalence of M1 over M2 subpopulation (Fig. 6D) was observed, suggesting a generally negligible inflammation of the hinge area.

#### Morphological evaluation

**Figure 7:**
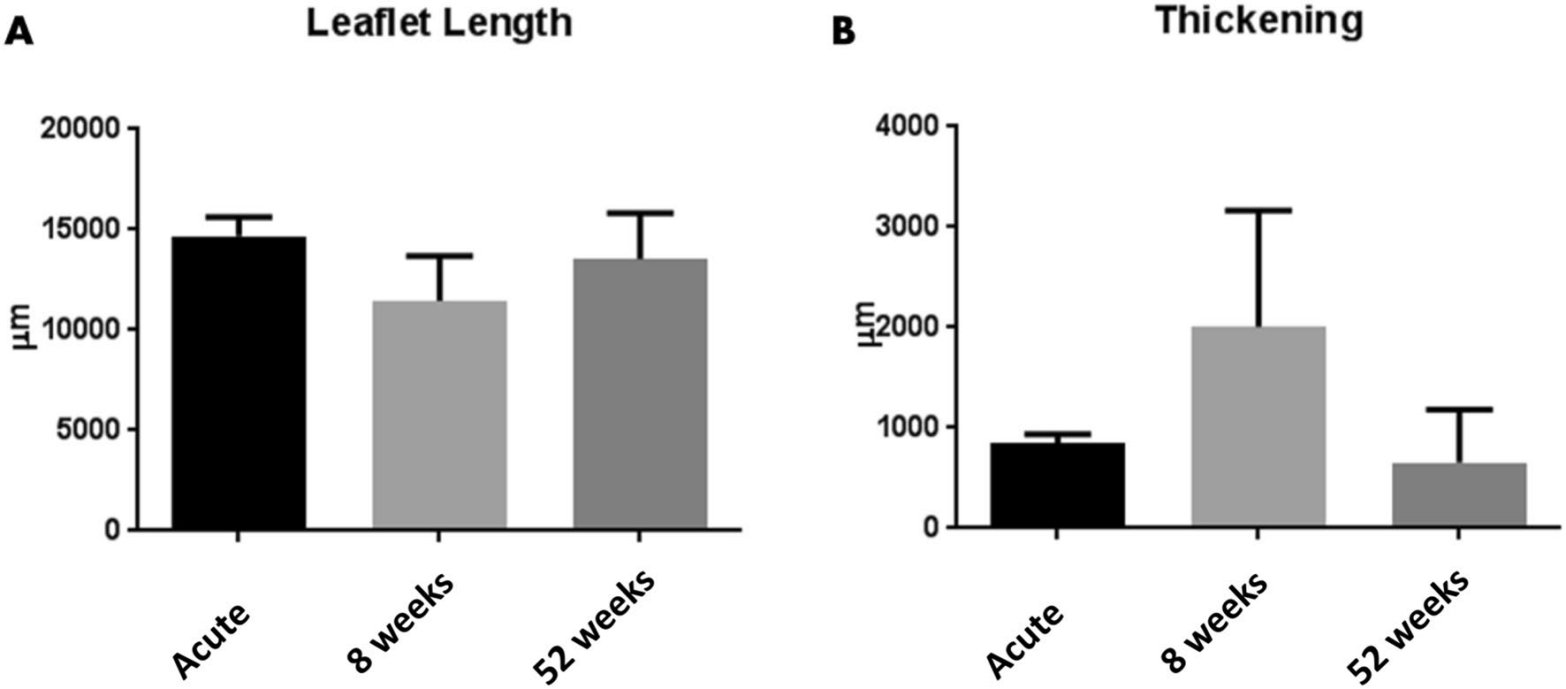
Tissue geometry of *second-generation* TEHVs. (**A**) Quantification of leaflet lengths from histological sections of *second-generation* TEHVs. (**B**) Quantification of hinge thickness from histological sections of *second-generation* TEHVs. Acute TEHVs represent the initial leaflet length and hinge thickness of acutely-implanted valves. Measurements are expressed as average value ± standard deviation.

Comparable to what was reported for *first-generation* THEVs, *second-generation* TEHVs displayed an initial 23% shortening of the leaflet length at 8 weeks. However, and in contrast to the *first-generation*, the shortening did not progress over time suggesting that an equilibrium of leaflet length was achieved in the 52 weeks tissue samples (Fig. 7A, Supplementary Table 2), which is an important parameter to ensure long-term valve performance^7^. In addition, the hinge region (Fig. 7B) showed a 52% increase of thickness at 8 weeks, which however normalized to the initial control values of the acute explants after 52 weeks (Fig. 7B, Supplementary Table 2).

## Discussion

Inflammation is an inescapable process, which naturally follows implantation of any device. Tissue-engineered implants designed for in-situ approaches aim at inducing a controlled inflammatory response to ultimately enable orchestrated healing and remodeling cascades^26^. Indeed, the impact of host cells on the ongoing inflammatory response and remodeling process changes over time and strongly depends on the type of implanted biomaterial, its geometry, and also on the overall hemodynamic conditions to which it is subjected^25,27–30^.

**Figure 8:**
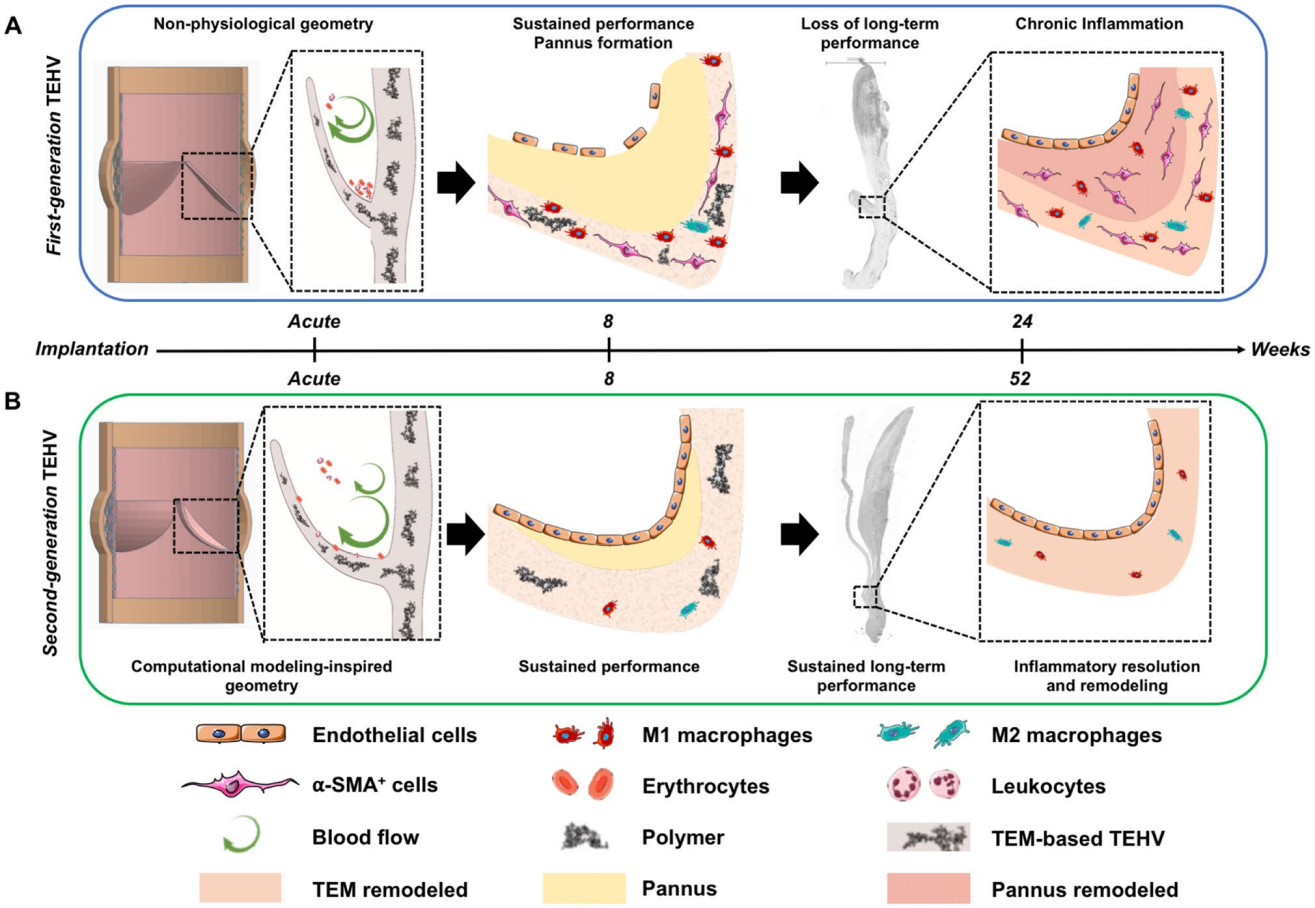
Summary of host cell infiltration behavior in response to the geometries of *first-* and *second-generation* TEM-based TEHVs. (**A**) We hypothesize that *first-generation* TEHVs presented a non-physiological geometry, which hindered the physiological formation of blood vortexes (arrows), thereby favoring the deposition of erythrocytes and leukocytes in the hinge region shortly after implantation. At 8 weeks follow-up, *first-generation* TEHVs showed sustained short-term performance with high expression of M1 macrophages and α-SMA^+^ cells markers. These inflammatory cells were mostly localized in the implanted TEM material, while few to no cells were detected in the newly formed pannus at the hinge area. Endothelialization was incomplete at this stage. After 24 weeks follow-up, *first-generation* TEHVs exhibited moderate to severe insufficiency, with a chronic inflammation and extensive maladaptive remodeling of the TEM material and pannus. This maladaptive remodeling was associated with evident hinge region thickening, abundant M1 and M2 macrophages, and α-SMA^+^ cells expression. Image was created using the software Tinkercad under *Creative Commons license Non-Commercial 3.0* and Servier Medical Art under *Creative Commons license 3.0*. (**B**) *Second-generation* TEHVs presented a computational modeling-inspired geometry, which allowed a more physiological performance of the TEHVs after implantation, thereby possibly favoring vortex formation and blood wash-out from the hinge area. After 8 weeks, *second-generation* TEHVs displayed sustained performance, with few M1 macrophages and negligible presence of M2 and α-SMA^+^ cells in the hinge region. At this timepoint, endothelialization of the valve surfaces, and in particular, of the hinge area was complete. At 52 weeks, *second-generation* TEHVs still exhibited excellent performance, with a similar cellular composition to the 8 weeks timepoint, comprising M1 macrophages and negligible presence of M2 and α-SMA^+^ cells. This inflammatory response highlighted the resolution and adaptive positive remodeling by the host towards *second-generation* TEM-based TEHVs. Image was created using the software Tinkercad under *Creative Commons license Non-Commercial 3.0* and Servier Medical Art under *Creative Commons license 3.0*.

In this study, we investigated the inflammatory host cell response in two different generations of TEM-based TEHV explants, which have been previously implanted as pulmonary valve replacements in a translational sheep model^6,7^. To better understand that, we performed an independent, in-depth qualitative and quantitative (immuno-)histological tissue analysis focusing on the key cellular players of the inflammatory and remodeling processes comprising M1 and M2 macrophages, α-SMA^+^ cells, lymphocytes, and ECs. In agreement with previously published studies^6,7^, we report the active role of inflammatory and immune cells in both the regenerative and the maladaptive remodeling of TEM-based TEHVs. Our results indicate that for *first-generation* TEHVs, the abundance of macrophages in the hinge area is associated with a considerable number of lymphocytes and α-SMA^+^ cells and resulting tissue fibrosis. To the contrary, the physiological-like geometry of *second-generation* TEHVs resulted in a milder inflammation with a reduced presence of macrophages, lymphocytes, and α-SMA^+^ cells. These results highlight how a milder inflammatory response (represented by a limited number of macrophages, lymphocytes, and α-SMA^+^ cells in the tissue) resulting from a more physiological-like geometry might elicit an adaptive remodeling of the implanted TEM-based TEHV. On the other hand, a strong inflammatory response with abundance of macrophages, as observed for the simplified *first-generation* TEHV geometry, can cause a maladaptive tissue remodeling, with leaflet thickening and shortening. Our main findings have been summarized in Fig. 8, where we highlight the differences in valve performance, in leaflet remodeling and, importantly, in the presence of M1 and M2 macrophages, α-SMA^+^ cells, and ECs in *first-* or *second-generation* TEHVs depending on the adopted geometry.

### Maladaptive remodeling of first-generation TEHVs

α-SMA^+^ cells have been repeatedly reported as a key cell type involved in leaflet shortening and retraction of TEHVs^8,31,32^, however their exact role has never been fully elucidated so far. In this study, we have identified two potential regulators that may play an important role in the infiltration and/or differentiation of α-SMA^+^ cells in the hinge area of our *first-generation* TEHVs: macrophages and ECs.

In *first-generation* valves, the chronic inflammatory response was confirmed by a general abundance of M1 over M2 macrophages, and an overall greater number of macrophages and lymphocytes when compared to *second-generation* TEHVs. The ongoing chronic inflammation may be explained by the presence of polymeric scaffold remnants until the 24 weeks follow-up, that are characterized by surrounding fibrous tissue, eosinophils, macrophages, multinucleated giant cells and lymphocytes (Fig. 2), thus impacting on the degree of inflammation of the host towards the implant.

Macrophages are known to actively impact on the degree and severity of damage and fibrosis by recruiting α-SMA^+^ cells to the inflammation site^33^, and by the transition from macrophages to myofibroblasts^34^. Macrophages are responsible for the release of pro-inflammatory cytokines and metalloproteinases that enhance the inflammatory response and influence the in-vivo wound healing by contributing to myofibroblasts proliferation and recruitment^35^. Briefly, in response to a tissue injury, macrophages first express the pro-inflammatory M1 phenotype, releasing TNF-α, IL-1β, IL-12, and IL-23. Subsequently, M2 macrophages take over by secreting IL-10 and TGF-β to reduce the inflammation^36^. During this process, macrophages express a complex phenotype, where cell polarization changes from M1 to M2 over time^37^, thereby actively regulating fibrotic tissue formation and wound healing^38^. In the presented study, we observed a steady presence of M1 macrophages, associated to an increase in M2 macrophages over time in *first-generation* TEHVs. Interestingly, M2 macrophages have been repeatedly reported to also influence fibrosis, by undergoing macrophage-to-myofibroblast transition^34^, by promoting the fibrogenic activities of fibroblasts^39^, and by inducing α-SMA expression in fibroblasts^40^. In addition, in-vitro experiments co-culturing macrophages and α-SMA^+^ cells demonstrated reduced apoptotic rates for both cell types, suggesting a beneficial interaction which promotes mutual survival^41^.

The strong inflammatory response and the presence of α-SMA^+^ cells observed in *first-generation* TEHVs can be associated to the high strain profile exerted on the matrix as well as to the incomplete endothelialization at the earliest time points. Physiologically, changes in mechanical milieu can influence cell differentiation mechanisms, matrix remodeling, and the endothelialization process, and are often correlated to valvular pathologies^25^. Native valvular ECs are indeed responsible for the anti-thrombogenic properties, as well as for the retention of the quiescent state of valvular interstitial cells^42,43^. We may therefore hypothesize that the lack of ECs has caused the activation of the infiltrated interstitial cells towards contractile α-SMA^+^ cells during the early stages of TEHV remodeling^42,44–46^. In line with our (immuno-)histological findings, we also observed leaflet shortening, hinge area thickening, and wall-leaflet fusion phenomena of *first-generation* TEHVs. These adverse remodeling events were assumed to be mainly triggered by the presence of contractile αSMA^+^ cells and may be a consequence of the simplified TEHV geometry, comprising a non-physiological hinge design. This may have contributed to the continuous thrombus deposition and pannus formation at the hinge region, which may have finally hindered the development of a confluent endothelium during the first 8 weeks after implantation. Notably, at every time point considered in our present analysis, the hinge region of *first-generation* TEHVs presented with an abundant amount of inflammation-associated cells (i.e.: leukocytes, eosinophils, macrophages, multinucleated giant cells, and α-SMA^+^ cells) which are involved in the formation of the pannus and the associated fibrotic tissue^33^ (Fig. 8A).

As previously reported, *first-generation* TEHVs showed good early recellularization, self-repair capacity, and good valve performance within the first 8 weeks in-vivo. However, mild to moderate insufficiency was first observed at 16 weeks, and valvular performance gradually worsened over time, with moderate-to severe insufficiency at 24 weeks, which was mainly caused by wall/leaflet fusion phenomena^6^ (Fig. 8A).

Taken together, the results of the present study extend our understanding of the maladaptive remodeling observed in our *first-generation* TEHVs. As highlighted by Fig. 8A, our findings support the hypothesis that the extended presence of M1, combined with the increasing number of M2 macrophages over time and the lack of a confluent endothelium in *first-generation* TEHVs significantly impacted valve remodeling by inducing myofibroblast differentiation, causing pannus formation and subsequent increase in the hinge thickness during the first remodeling phases.

### Second-generation TEHVs, towards a native-like remodeling

To date, independently of the tissue engineering technology used^6,47–53^, several studies—among which also our *first-generation* TEHV—reported a gradual loss of TEHV performance within few months after implantation, due to maladaptive tissue remodeling (thickening, leaflet shortening). However, here we show that the tissue analysis of our *second-generation* TEHVs did not reveal signs of pannus formation nor thickening of the hinge area. Importantly, *second-generation* TEHVs presented with much less leaflet shortening when compared to their *first-generation counterparts*. Notably, it appears that both TEHVs generations undergo an early shortening within the first 8 weeks, which then continues in the *first-generation* valves leading to functional failure (negative remodeling-driven continuous shortening), while the leaflet length remains completely stable and unchanged over time in the second-generation TEHVs, thereby maintaining an excellent performance profile (functional remodeling).

In fact, the improved and more physiological geometry of *second-generation* TEHVs is not only beneficial from a mechanical point of view, but also from an inflammatory host cell response perspective (Fig. 8B). Indeed, the long-term performance demonstrated by our *second-generation* TEHVs^7^ was characterized by an overall low inflammatory cell infiltration, negligible amount of macrophages, and, importantly, the absence of α-SMA^+^ cells in the hinge region at all the time-points considered (acute, 8 weeks, and 52 weeks). This, in relation to the complete endothelialization of the hinge area already at 8 weeks after implantation, resulted in an overall milder tissue inflammation (Fig. 8B).

Finally, this controlled inflammatory response is reflected by the observation of positive tissue remodeling outcomes. In this regard, we previously demonstrated that, similarly to a native valve, our computational modeling-inspired valve geometry can better sustain strain on the valve leaflets, thereby ensuring a more physiological long-term performance^7^. Indeed, one of the major causes of leaflet shortening has been identified in leaflet compression. While *first-generation* TEHVs showed an overall compression of the leaflet, from the coaptation to the hinge region, *second-generation* TEHV geometry could efficiently limit leaflet compression to just the coaptation area^8^. Second, we did neither detect thickening nor pannus formation in the hinge area. We may therefore hypothesize that *second-generation* TEHV geometry seem to better mimic native leaflet anatomy in the hinge area, thereby promoting the wash-out of fibrin deposits and preventing blood stagnation (Fig. 8B). The resulting favorable hemodynamics may therefore have enabled long-term performance of such *second-generation* TEHVs^7^.

#### 8 weeks in-vivo follow-up, a turning point in the TEHV remodeling process?

As highlighted by our findings both generations of TEHVs presented with promising cellular infiltration and self-repair (remodeling) potential up to 8 weeks in-vivo. However, it is important to recognize that the remodeling process of *first-* and *second-generation* TEHV progresses into two distinct directions beyond 8 weeks (Fig. 8A,B). So far, the relevance of TEHV geometry was mainly considered on a mechanical^8^ and/or functional^7^ level. In the present study, we demonstrate that the TEHV geometry also strongly influences the outcome on a host cell response level (Fig. 8A,B).

At 8 weeks post implantation, *first*- and *second-generation* TEHVs showed comparable remodeling of the leaflet, with similar values of leaflet lengths (23% vs. 19% shortening for *first-* and *second-generation,* respectively) and hinge thicknesses. To the contrary, the differences observed in the number, phenotype, and distribution of infiltrating cells was substantially different underscoring the importance and impact of TEHV geometry. On one hand, *first-generation* TEHVs showed abundant cell infiltration, with approximately 20 times more cells per μm^3^ than *second-generation* TEHVs at the same time point. Next, abundance of inflammatory cells, such as eosinophilic cells, M1 macrophages, α-SMA^+^ cells, together with lack of a confluent endothelium in the hinge area were detected.

On the other hand, computational modeling-inspired *second-generation* TEHVs demonstrated an overall lower cellular infiltration with the negligible presence of M1/M2 and α-SMA^+^ cells already 8 weeks after implantation. Remarkably, endothelialization of the hinge region was complete already at this time point. Hence, despite a comparable leaflet morphology, our qualitative and quantitative cell analysis indicates a substantial difference between the valves already at 8 weeks that resulted in two distinct remodeling outcomes (maladaptive *first-generation* TEHVs versus adaptive and functional in *second-generation* TEHVs) beyond 8 weeks. This finding highlights the importance of early host cell response in controlling long-term valvular remodeling, and provides important insight on time and type of cells that could be included in in-silico models to further enhance the outcome on tissue remodeling prediction^9^.

## Limitations

This study is an in-depth evaluation of TEHV explants obtained from previously performed in-vivo transcatheter implantations of two independent studies^6,7^. Consequently, the available time points and number of samples were limited and unpaired between the experiments (Table 1). Hence, the possibility to perform statistical analyses to compare *first-* and *second-generation* THEVs was limited. Despite this, we showed that both TEHVs generations underwent an initial leaflet shortening at 8 weeks follow-up. However, *first-generation* TEHVs experienced a progressive leaflet shortening, leading to a 70% reduction in length at 24 weeks post-implantation. To the contrary, leaflet shortening for *second-generation* TEHV did not progress over time thereby enabling the TEHV to remain functional for up to 1 year. This result is in line with what was previously reported for *second-generation* TEHVs^7^, even though a direct comparison between these studies is not possible because of 1) the different amount of samples used to evaluate the leaflet length (11^7^ vs 3 valve explants) and 2) the different analysis techniques adopted to measure the leaflet length (caliper measurement^7^ vs. histology section measurement). In this regard, it has been showed that a collagenous tissue processed for histology (i.e.: using formaldehyde fixation and paraffin wax embedding) can be subjected to a 33% shrinkage^54^. Our study might therefore also highlight the importance of choosing the most appropriate measurement strategy to precisely report explanted TEHV remodeling measurements. In addition, immunohistochemistry proved to be a suboptimal technique to evaluate the presence of ECs due to cutting artefacts that may have caused cell detachment and lack of visible staining. In this regard, future studies should also better assess the integrity and functionality of the ECs layer, as well as evaluate the presence of activated platelets and microvesicles in the plasma. In this regard, analyses of anticoagulation and anti-thrombotic properties of ECs should be carefully considered when planning future in-vivo studies.

Next, we here focused our analysis on the hinge area of the TEHVs that, due to its important load-bearing activity, is subjected to high deformations^24^. Indeed, mechanical strain is a well-known regulator of cell behavior and differentiation^25^, and can therefore impact the (mal)adaptive remodeling phenomena in TEHVs. However, future in-vivo studies should consider additional time points as well as different TEHVs regions (e.g. the leaflets), to assess the first stages of inflammation (3–4 weeks follow up) and the resolution of the inflammatory status (12–24 months).

Finally, future studies should investigate how TEHV geometry will impact on the mechanical and hemodynamic valvular environment, also taking into consideration blood flow patterns through the valve, to better understand the mechanobiology of valvular remodeling. In this context, in-depth understanding of the molecular pathways underlying the complex interactions between inflammatory host cells, matrix remodeling, and scaffold degradation processes over time, may be crucial to better predict or even to anticipate (mal)adaptive remodeling phenomena.

## Conclusions

This study highlights the strong interplay between TEHV geometry and its related inflammatory host cell response, which ultimately drives the tissue remodeling outcomes in TEHVs. *First-generation* TEHVs, with a simple valve-geometry, experienced a chronic inflammatory response characterized by abundant presence of M1 and M2 macrophages and α-SMA^+^ cells, which finally led to leaflet retraction and valve insufficiency. In contrast, *second-generation* TEHVs displayed an overall lower inflammatory response with only few M1 and M2 macrophages and α-SMA^+^ cells being present. These results highlight how an analytical, computational-inspired TEHV geometry does not only impact the mechanical strain distribution on the leaflets which is critical for valve performance, but also how it orchestrates host cell response towards functional remodeling, thereby enabling long-term performance of a TEHV. Our findings further provide important insight into the (inflammatory) host cell response and the related remodeling processes of TEM-based TEHVs which represents a key requisite for the safe and successful clinical translation of such technologies.

## Methods

In this study, we performed a pooled, in-depth analysis of TEM-based TEHV tissue explants (n = 20) from our previously reported in-vivo studies^6,7^, as well as from additional unpublished data. The ethics committee and local authorities (Veterinäramt, Gesundheitsdirektion, Kanton Zürich [197/2010], Switzerland and the Regional Office for Health and Social Affairs Berlin, LAGeSo, Berlin; approval.

no. G0111/11) approved the previously reported in-vivo studies^6,7^, which were performed in compliance with the Guide for the Care and Use of Laboratory Animals, published by the National Institutes of Health (NIH publication No. 85–23), as well as the guidelines of the European and German societies of Laboratory Animal Science (FELASA, GV-SOLAS) and the ARRIVE guidelines (Animal Research; Reporting of in-vivo Experiments). Detailed information about the manufacturing, in-vitro characterization, and in-vivo testing of *first-*^5^ and *second-generation*^7^ TEHV have been previously reported^6,7^. Compared to *first-generation* TEHVs^6^, the use of an insert^8^ to impose a computational modelling-inspired valve geometry was the only difference in the manufacturing protocol of *second-generation* TEHVs^7^.

According to the follow-up schemes of our in-vivo studies^6,7^, a total of fifteen TEHV tissue samples (n = 15) were obtained from explants at the following time points: acute (considered as control), 8 weeks, 16 weeks, 24 weeks, and 52 weeks (1 year). The overview of available samples is reported in Table 1. In addition, the tissue samples for the acute (n = 2) and 8 weeks time point (n = 3) of the *second-generation* valve were obtained from an independent pilot series of animals (n = 5) which has not been published before (unpublished data), and were produced using the same culture conditions. Qualitative and quantitative analyses have been performed as described below to evaluate the inflammatory host cell response to the implanted TEHVs particularly in the hinge region of the leaflets, as it acknowledged to be the most subjected area to maladaptive remodeling phenomena.

### Qualitative tissue analysis

Samples from explanted TEHVs (Table 1) were analyzed qualitatively to characterize the presence of inflammatory host cells using immunofluorescence, histology, immunohistochemistry, and scanning electron microscopy (SEM) techniques.

#### (Immuno)histochemistry and immunofluorescence stainings

In brief, TEHVs samples were fixed in formalin, embedded into paraffin, and cut consecutively into 5 µm slices with a sliding microtome at room temperature. The specimens were deparaffinized by treatment with xylene followed by a series of decreasing concentrations of ethanol (100–50%). Cellular infiltration and tissue appearance were evaluated by using Haematoxilin and Eosin (H&E) and Masson Goldner (MG) staining, and CD31, von Willebrand Factor (VWF), and α-SMA staining. The slides were visualized by Zeiss Mirax Midi microscope (Carl Zeiss Microimaging) and assessed with Pannoramic Viewer (3DHistech).

To characterize the phenotype of the infiltrated cells within the explanted TEHVs, immunofluorescence was performed following antigen retrieval using citrate (10 mM citric acid at pH 6.0) which was then brought to boiling temperature (96 °C) for 20 min. Primary antibody for CCR7 (M1 marker) and CD163 (M2 marker) were incubated for over-night at 4 °C. After washing with PBS, secondary antibodies were applied for 2 h (Supplementary Table 1). Cell nuclei were counterstained with DAPI. The stained sections were visualized using a fluorescence slide scanner (ZEISS Axio Scan.Z1, Carl Zeiss AG). Image processing was performed using the Axio Scan.Z1 ZEN lite imaging software (Blue version, 2012; Carl Zeiss AG).

#### Scanning electron microscopy (SEM)

SEM was used to assess and confirm the degree of endothelialization for the two TEHVs geometries^6,7^. For further details see the Supplementary Material section.

### Quantitative tissue analysis

#### Inflammatory host cell infiltration

Cellular infiltration was quantified automatically and manually with ImageJ software^55^ in three pictures across the hinge area and in two consecutive sections for a total thickness of 10 µm (20 × magnification; area comprising 420,000 µm^2^ per picture). Pictures of stained sections were converted into grey-value images (16-bit). Quantification was performed by counting the cells positive for CCR7, CD163, or a combination of both (double stained cells) and compared to the total number of cells, detected via DAPI. The total number of cells infiltrating the wall region was used as control value for the complete cellular infiltration in the hinge area.

#### Morphological evaluation

Hinge thickness and leaflet length were measured manually for both *first-* and *second-generation* TEHVs using the Panoramic Viewer software (3DHistech, Ltd.). For both TEHVs geometries, hinge thickness was measured in two different sections of 5 μm each, and in three locations per section, for a total of 6 measurements. Leaflet length was measured in two different sections of 5 μm each, by using the highest number of segments possible to increase the accuracy of the measurements. Analyzed samples comprised the following follow-up time-points: acute, 8 weeks, 16 weeks, 24 weeks, and 52 weeks (Table 1).

#### Statistics

Measurements are represented as mean value ± standard deviation unless stated otherwise. Leaflet length and hinge thickness measurements were averaged for each sample (Supplementary Table 2).

## Supplementary information

Supplementary Information.

## Data Availability

Data are available upon request and completion of a Materials Transfer Agreement (MTA).
