# Supplementary material for: Geometry influences inflammatory host cell response and remodeling in tissue-engineered heart valves in-vivo
Source: Sci Rep. 2020 Nov 16;10:19882. doi: 10.1038/s41598-020-76322-9 (PMC7669851; doi:10.1038/s41598-020-76322-9)
Supplement: Supplementary file 1 — Supplementary Information. [file 41598_2020_76322_MOESM1_ESM.pdf]

Geometry influences inflammatory host cell response and remodeling in tissue-engineered heart valves in vivo

Sarah E. Motta [1,2,9], Emanuela S. Fioretta[1,9], Valentina Lintas[2], Petra E. Dijkman[1], Monika Hilbe[3], Laura Frese[1], Nikola Cesarovic[4,5], Sandra Loerakker[6,7], Frank P. T. Baaijens[6], Volkmar Falk[4,5,8] Simon P. Hoerstrup[1,2], Maximilian Y. Emmert[1,2,4,8,*]

[1] Institute for Regenerative Medicine (IREM), University of Zurich, Zurich, Switzerland

[2] Wyss Translational Center Zurich, University and ETH Zurich, Zurich, Switzerland

[3] Institute of Veterinary Pathology, University of Zurich, Zurich, Switzerland

[4] Department of Cardiothoracic and Vascular Surgery, German Heart Center Berlin, Berlin, Germany

[5] Department of Health Sciences and Technology, Swiss Federal Institute of Technology, Zurich, Switzerland

[6] Department of Biomedical Engineering, Eindhoven University of Technology, Eindhoven, The Netherlands

[7] Institute for Complex Molecular Systems, Eindhoven University of Technology, The Netherlands

[8] Department of Cardiovascular Surgery, Charité Universitätsmedizin Berlin, Berlin, Germany

[9]These authors contributed equally and share first authorship

***Corresponding author:**

Maximilian Y. Emmert, MD, PhD
Institute for Regenerative Medicine (IREM), University of Zurich, Wagistrasse 12, 8952 Schlieren, Switzerland. Tel.: +41 44 634 5610, E-mail: maximilian.emmert@irem.uzh.ch

Material and Methods

Scanning electron microscopy (SEM)

SEM was used to assess and confirm the degree of endothelialization for the two TEHVs designs (JACC, Emmert). Briefly, samples were fixed in 2% glutaraldehyde (Sigma-Aldrich; Switzerland), dehydrated with a sequence of different EtOH concentrations, and embedded in a solution of EtOH with increasing concentration of plastic components. Sections were then cut in an ultramicrotome and finally platinum-sputtered for imaging.

Supplementary Table 1: Summary of the antibodies used for immunofluorescence.

First-generation **TEHV**	**Primary AB**	**Secondary AB**
**M1**	Rabbit anti-CCR7 (Abcam, ab32527)	Goat anti-rabbit Alexa 568
**M2**	Mouse anti-rat CD163 (Abd serotec, MCA342GA)	Goat anti-mouse Alexa 488
Second-generation **TEHV**	**Primary AB**	**Secondary AB**
**M1**	Rabbit anti-CCR7 (Abcam, ab32527)	Goat anti-rabbit Alexa 568
**M2**	Mouse anti-rat CD163 (Abd serotec, MCA342GA)	Goat anti-mouse Alexa 488

Supplementary Table 2: *First-* and *second-generation* TEHVs leaflet lengths and hinge thickness measurements. Measurements are represented as mean value ± standard deviation

**Follow-up [weeks]**	***First-generation*** **TEHV**	
	**Leaflet length [µm]**	**Hinge thickness [µm]**
**Control**	11533.33 ± 1042.43	754.17 ± 12.81
**8**	9365.83 ± 273.14	1686.67 ± 784.39
**16**	6597.16 ± 1703.23	2715 ± 700.72
**24**	3612.916 ± 1848.99	2255.83 ± 355.41
	Second-generation **TEHV**	
	**Leaflet length [µm]**	**Hinge thickness [µm]**
**Control**	14451.43 ± 1081.86	870.57 ± 107.34
**8**	11415.55 ± 2017.84	1996.67 ± 1001.77
**52**	13538.33 ± 2014.32	651.33 ± 445.42

Results

Host cellular infiltration in *first-generation* TEHVs

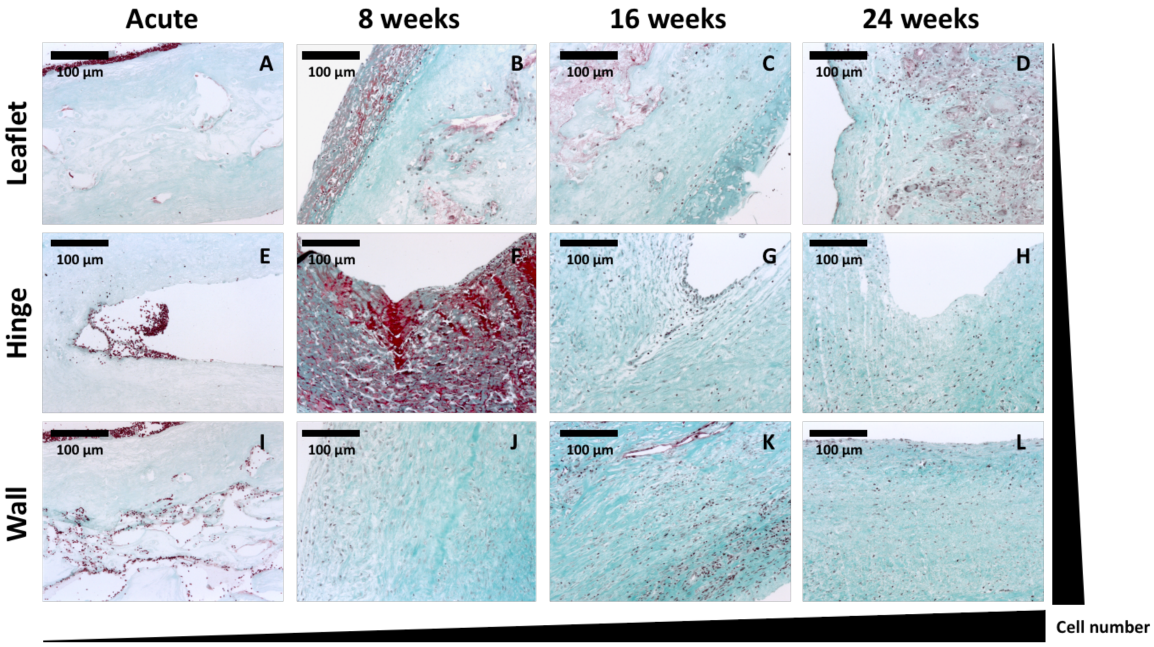

Supplementary Figure 1: Representation of endogenous cellular infiltration in *first-generation* TEHVs. Host cells gradually repopulate TEHVs over time starting from the wall and progressively migrating towards the hinge and the leaflet area (scale bars 100 µm).

Endothelialization of *first-generation* TEHVs

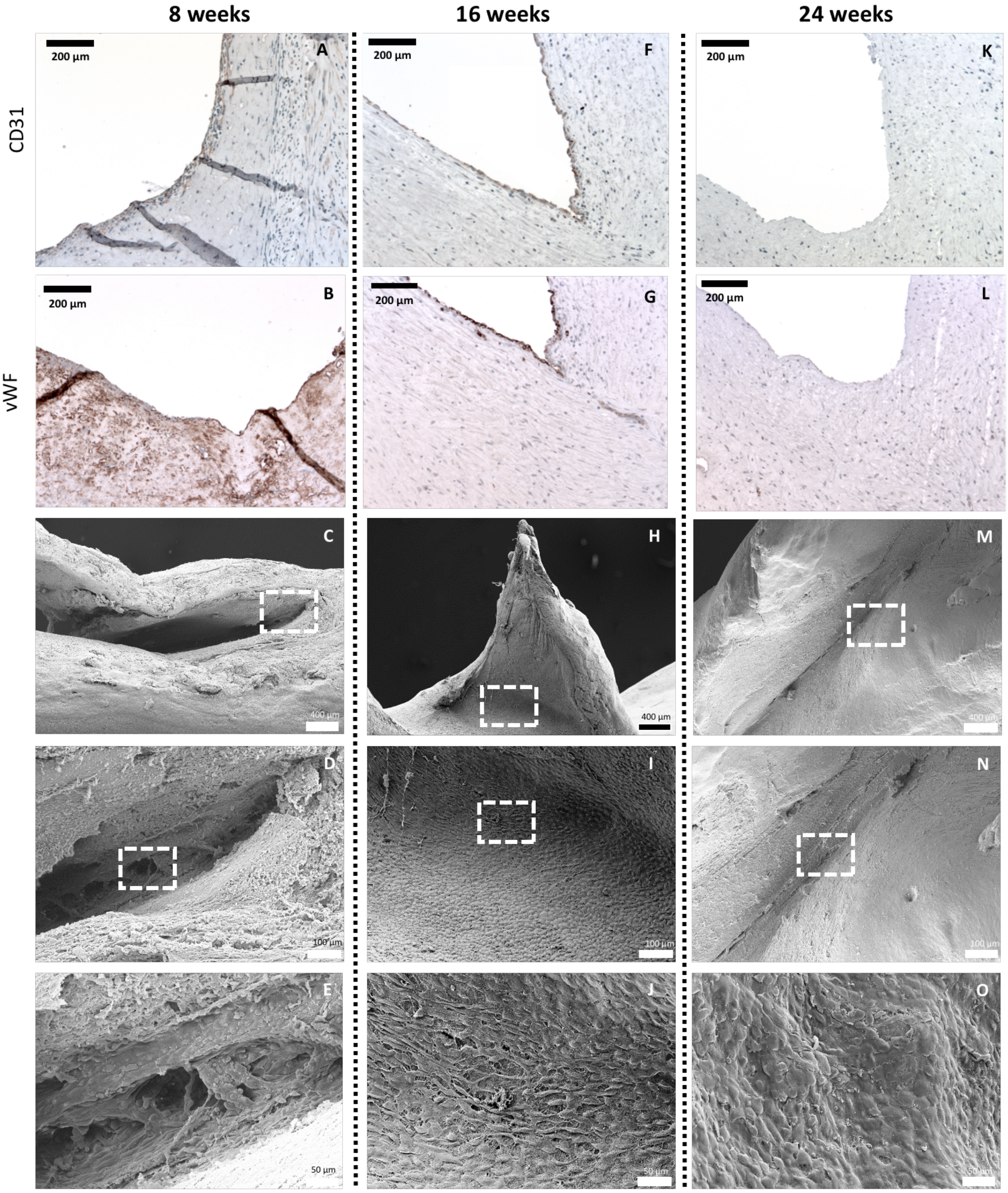

Supplementary Figure 2: Assessment of endothelialization of *first-generation* TEHVs via immunohistochemistry and SEM imaging. Images of the TEHVs hinge area stained for CD31 and vWF at different time-points (8, 16, and 24 weeks) (scale bars 100 µm). SEM images of the hinge area of at different time-points (8, 16, and 24 weeks) and at three different magnifications. Endothelial cells are visible already at 8 weeks, but they form a confluent layer only at later time-points.

Host cellular infiltration in *second-generation* TEHVs

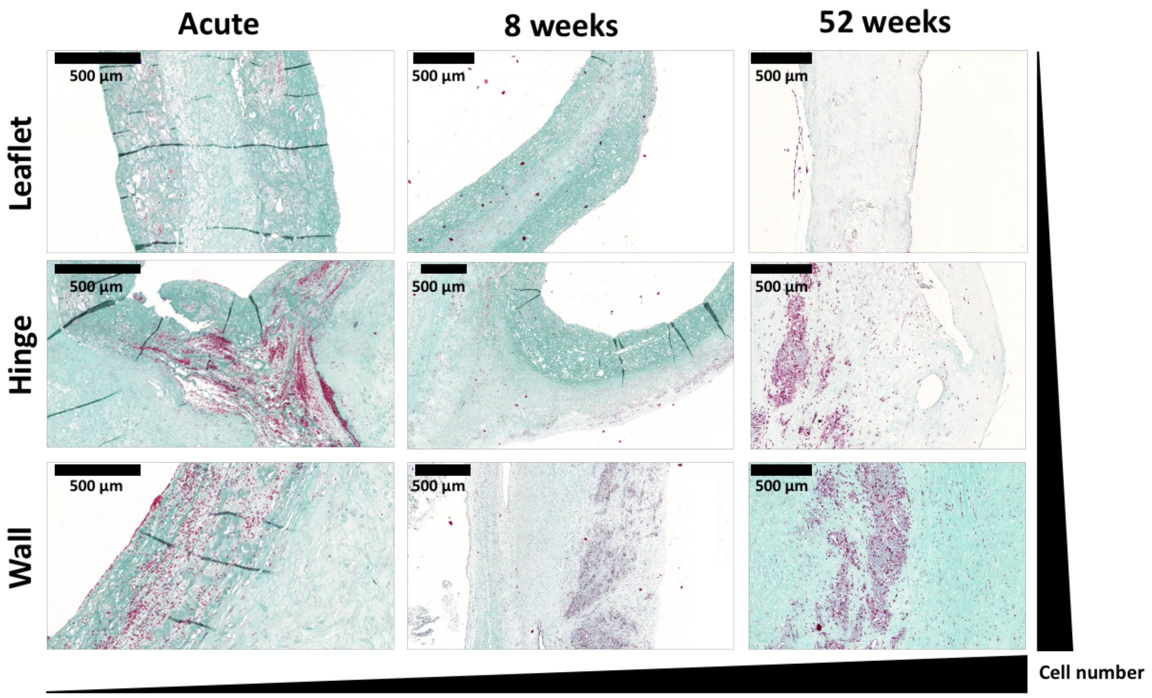

Supplementary Figure 3: Representation of endogenous cellular infiltration in *second-generation* TEHVs. Host cells gradually repopulate TEHVs over time starting from the wall and progressively migrating towards the hinge and the leaflet area (scale bars 500 µm).

Endothelialization of *second-generation* TEHVs

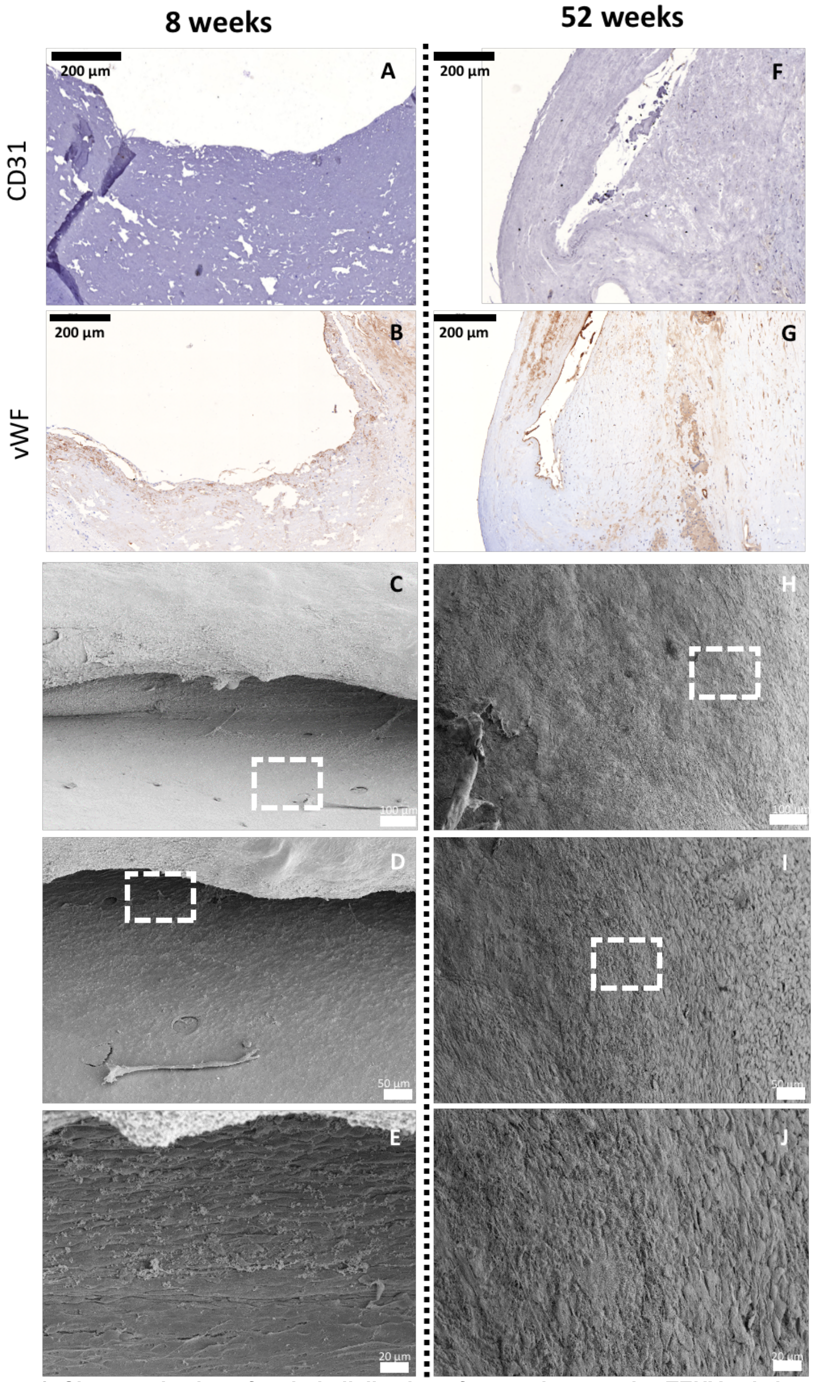

Supplementary Figure 4: Characterization of endothelialization of *second-generation* TEHVs via immunohistochemistry and SEM imaging. Images of the TEHVs hinge area stained for CD31 and vWF at different time-points (8 and 52 weeks). Endothelial cells are visible and lining the surface of TEHVs. Cutting artefacts might prevent the correct representation of endothelial cells presence at 8 and 52 weeks for the CD31 staining (scale bars 200 μm). SEM images of the hinge area of *second-generation* TEHVs at different time-points (8 and 52 weeks) and at different magnifications. A confluent and oriented endothelium is already visible at 8 weeks and retained also after 52 weeks in-vivo.
